# Effect of Clonidine Premedication on Blood Loss in Spine Surgery

**DOI:** 10.5812/aapm.2197

**Published:** 2012-04-01

**Authors:** Zahra Taghipour Anvari, Nader Afshar-Fereydouniyan, Farnad Imani, Mojgan Sakhaei, Babak Alijani, Masood Mohseni

**Affiliations:** 1 Department of Anesthesiology and Pain Medicine, Rasoul-Akram Medical Center, Tehran University of Medical Sciences (TUMS), Tehran, Iran; 2 Department of Neurosurgery, Rasoul-Akram Medical Center, Tehran University of Medical Sciences (TUMS), Tehran, Iran

**Keywords:** Clonidine, Anesthetics, Blood Loss, Surgical, Prevention and Control, Hypotension, Bloodless Medical and Surgical Procedures, Adrenergic Alpha-2 Receptor Agonists, Spinal Fractures

## Abstract

**Background:**

Blood loss in spine surgery is an important issue, even though it has been understudied compared with hip and knee arthroplasty.

**Objectives:**

In this study, we evaluated the effect of oral clonidine as premedication on blood loss in lumbar spine fusion surgery under anesthesia with propofol and remifentanil.

**Patients and Methods:**

In this double-blind, randomized clinical trial, 30 patients who were undergoing lumbar spine posterior fusion surgery due to traumatic fracture were allocated randomly into 2 groups. The study group (clonidine group) received a 200-μg oral clonidine tablet 60–90 minutes before anesthesia, and the control group received placebo at the same time. Induction and maintenance of anesthesia and the mean target arterial pressure for controlled hypotension with remifentanil were the same in the 2 groups. We compared the amount of intraoperative blood loss, dose of remifentanil/hour administered, need for nitroglycerine to reach the mean target arterial pressure when remifentanil was insufficient, duration of operation, and surgeon’s satisfaction of a bloodless field between groups.

**Results:**

There was no statistically significant difference between groups in age (*P* = 0.115), sex (*P* = 0.439), weight (*P* = 0.899), operation time (*P* = 0.2), or American Society of Anesthesiologists physical status score (*P* = 0.390).

Intraoperative blood loss and remifentanil dose administered per hour in the clonidine group were significantly less than in the control group (P = 0.002 and P = 0.001, respectively), but there was no significant difference in surgeon’s satisfaction between groups (P = 0.169).

**Conclusions:**

As an oral premedication, clonidine can reduce surgical blood loss in lumbar spine posterior fusion surgery, even at the same levels of mean arterial pressure (MAP) with the control group. Its use can be studied in more complicated spine surgeries, such as scoliosis and spinal deformity surgeries.

## 1. Background

Spinal fusion surgery is often associated with major blood loss, which is sometimes significant, requiring the transfusion of blood or blood products (1). Blood loss can be an acute problem not only in major deformity surgery but also in less extensive fusion procedures. Decreasing bleeding is important to maintain a patient’s hemodynamic stability and improve the surgical field. In spine surgery, the latter aspect is especially important, due to the vicinity of major and highly fragile neurological structures. The surgeon’s comfort shortens the operating time, which further decreases bleeding (2). Decreased bleeding also reduces the need for the transfusion of blood products, thereby reducing the risk of complications, such as hemolytic and non-hemolytic reactions, acute lung injury, transmission of viral and bacterial infections, hypothermia, and coagulation disorders.

Blood-sparing techniques can be divided into two groups, based on their goals: they are aimed at decreasing the bleeding itself [hemodynamically (e.g., controlled hypotension, local vasoconstrictors, epidural blockade) or with chemical/biological agents (e.g., desmopressin, aprotinin, tranexamic acid, epsilon-aminocaproic acid, estrogens, bone wax, hemostatic “sponges,” fibrin sealants)] or at decreasing the need for homologous transfusion (e.g., acute hemodilution, planned autologous transfusion, cell-saving systems, erythropoietin).

Controlled hypotension has been used with success in orthopedic surgery. It is applied widely in spine surgery, and several studies have demonstrated it to be useful in spine surgery (3–6). Agents that are used alone to induce controlled hypotension include inhalation anesthetics, sodium nitroprusside, nitroglycerin, trimethaphan, alprostadil (prostaglandin E1), adenosine, remifentanil, and agents that are used in spinal anaesthesia. Agents that can be used alone or as adjuvants include calcium channel antagonists (e.g., nicardipine), beta-adrenoceptor antagonists (e.g., propranolol, esmolol), and fenoldopam. Agents that are primarily used adjunctively include angiotensin converting enzyme (ACE) inhibitors and clonidine. The preferred technique is a combination of remifentanil with propofol or an inhalation agent (isoflurane, desflurane, or sevoflurane) (7–11). Alpha-2 adrenergic agonists (clonidine and dexmedetomidine) have been used successfully as adjuvants, oral premedication, and intravenous infusion during anesthesia to induce controlled hypotension (12–20). Clonidine is an alpha-2 adrenergic agonist, which has been used as a centrally acting antihypertensive drug. Recent studies have demonstrated it to have sedative, anxiolytic (21), analgesic, and anesthetic-sparing (it reduces the dose of anesthetic and analgesics used intra- and postoperatively) effects, and it stabilizes the circulatory system and reduces perioperative stress response (22–24).

## 2. Objectives

In this study, we used clonidine as oral premedication and as an adjuvant to remifentanil to induce controlled hypotension during posterior fusion of the lumbar spine and compared its effects in reducing intraoperative bleeding with remifentanil alone.

## 3. Patients and Methods

### 3.1. Patient Selection

We studied 30 patients aged 20–65 years with American Society of Anesthesia (ASA) physical status I–II who were candidates for posterior fusion of the lumbar spine. The exclusion criteria were significant underlying disease (hypertension, hepatic or renal disease, coagulation defects, diabetes mellitus); receiving beta-blockers, calcium channel blockers, dioxin, tricyclic antidepressants, anticoagulants, or clonidine; drug or alcohol abuse, and allergy to any of the drugs used in the study. Sample size was calculated per previous studies, considering α = 0.05 and statistical power of 90%, mean bleeding in the control group = 432 mL (standard deviation [SD] = 225), and mean bleeding in the clonidine group = 206 mL (SD = 106), which established a sample size of 13 for each group, calculated using STATA. Then, we studied 15 patients in each group.

### 3.2. Methods

In this prospective, randomized, double-blind study, a nurse randomly allocated 30 patients to 2 groups after obtaining written informed consent. Ninety minutes before entering the operating room, patients in group 1 received 1 200-μg tablet of clonidine (nearly equal to 3 μg/kg in an adult patient), and those in group 2 received a pantoprazole tablet as placebo. The next steps in the operating room were the same for both groups and performed by an anesthetist and a technician who were unaware of the type of premedication. Patients in both groups received 7 mL/kg of Ringer’s solution before induction of anesthesia and were monitored by the same system for heart rate (HR) and mean arterial pressure (MAP) (invasive and noninvasive). Electrocardiography, pulse oximetry, end-tidal CO2, and urine output were also monitored. After intravenous premedication with 0.05 mg/kg midazolam and 3 μg /kg fentanyl, anesthesia was induced with 2 mg/kg propofol and 0.5 mg/kg atracurium, maintained with propofol 100 μg/kg/min and atracurium 10 mg every 30 minutes and a remifentanil infusion of 0.1 to 1 μg/kg/min, which was titrated to a target MAP of 60–70 mmHg. If a 1-μg/kg/min dose of remifentanil was not enough to induce the target hypotension, a nitroglycerine infusion was added to the regimen. All patients were positioned (in the prone position) and operated on by the same team, comprising a neurosurgeon and neurosurgery resident. Positioning of all patients was performed using the same system and type of rolls (chest and pelvic rolls, leaving the abdomen free). The mechanical ventilation setting for all patients was set as follows: tidal volume = 10 mL/kg, respiratory rate = 10/min, and inspiration/expiration ratio = 1.3; then, according to end-tidal CO2, only tidal volume changes were made to achieve normocarbia. All patients received 1 μg/kg fentanyl 30–45 minutes before the end of the operation. Intraoperative fluids for all patients included ringer lactate as maintenance fluid and normal saline for deficits and losses, including replacement for blood loss to a transfusion threshold of hemoglobin = 10 g/dL. Intraoperative blood loss was estimated, based on the volume of blood in the suction bottle and the number of the bloody gauze pads, by the same anesthesiologist for all patients, who was unaware of the study details. The following data were recorded for each patient: age, sex, weight, operation time, total dose of remifentanil used, dose of remifentanil used per hour, need for nitroglycerin infusion for induced hypotension, and surgeon satisfaction. Surgeon satisfaction with a bloodless field was evaluated as follows: good (minimal or no bleeding), intermediate (modest bleeding and impairment of operating condition), and bad (significant bleeding and impairment of operating conditions).

## 4. Results

Thirty patients were studied in 2 groups: clonidine (n = 15) and control (n = 15).

There was no statistically significant difference between groups with regard to age, sex, or ASA physical status (*P* = 0.115, *P* = 0.349, and *P* = 0.390, respectively) (*Table 1*). There was also no significant difference in weight or duration of surgery, both of which can affect surgical blood loss, between groups (*P* = 0.899 and *P* = 0.2, respectively) (*Tables 1 and 2*).

The clonidine group had significantly less intraoperative blood loss (422.3 ± 139 mL; *P = 0.002*) compared to the control group (749.2 ± 304.5 mL). The clonidine group had also less remifentanil use for keeping the MAP in the desired range for controlled hypotension than the control group (1.3 ± 0.5 mg/h *vs*. 2.5 ± 1 mg/h; *P =* 0.001).

Surgeon satisfaction for a bloodless field was good in 14 (93.3%) of patients in the clonidine group compared to 10 (66.7%) patients in the control group, but the difference was not statistically significant (*P* = 0.169).

There was no episode of severe bradycardia that caused hemodynamic instability or was not reversible with atropine in either group.

There was no need for nitroglycerin to maintain controlled hypotension in either group.

**Table 1.** Age, Sex, ASA physical Status, and Weight Results in the Two Groups

	Clonidine Group	Control Group	*P* value
Age, y, Mean ± SD	44 ± 13.9	36.4 ± 11.5	0.115
Sex, No. (%)			0.439
Male	9 (60)	11 (73.3)	
Female	6 (40)	11 (27.7)	
ASA ^a^ physical status			0.390
ASA I	13 (86.7)	10 (66.7)	
ASA II	2 (23.4)	5 (33.3)	
Weight, Kg, Mean ± SD	67.1 ± 14.5	68.9 ± 18.1	0.899

^a^ Abbreviation: ASA, American Society of Anesthesia

**Table 2.** Blood Loss, remifentanil Usage, Duration of Operation and Surgeon Satisfaction in the Two Study Groups

	Clonidine Group	Control Group	*P* value
Blood loss, mL, Mean ± SD	422.3 ± 139	749.2 ± 304.5	0.002
Remifentanil used,mg/h, Mean ± SD	1.3 ± 0.5	2.5 ± 1	0.001
Duration of operation, h, Mean ± SD	3.6 ± 0.6	3.9 ± 0.7	0.2
Surgeon’s satisfaction, No. (%)			0.169
Good	14 (93.3)	10 (66.7)	
Inter-mediate	1 (6.7)	4 (26.7)	
Bad	-	1 (6.7)	

## 5. Discussion

Blood sparing in spine surgery is important, but its techniques have been understudied compared to other orthopedic and surgical fields, with the current practice based more on beliefs than evidence (2).

Controlled hypotension is among the most widely used techniques for reducing blood loss in various types of surgery, and remifentanil has been used successfully to induce controlled hypotension and reduce intraoperative blood loss in various types of surgery, including spine surgery (8–11). In our study, oral clonidine premedication as an adjunct to remifentanil resulted in significantly less blood loss during posterior spine fusion. Clonidine reduced intraoperative blood loss at the same levels of blood pressure as the control group, as the remifentanil dose was adjusted in both groups to the same target MAP of 60 to 70 mmHg. This finding is similar to results by Okuyama *et al.*, who observed that clonidine and prostaglandin E_1_ reduce blood loss during paranasal sinus surgery without inducing hypotension. Our results are also consistent with Lee *et al.*, noting differing paraspinal muscle blood flow at the same levels of hypotension with various drugs; thus, it appears that different drugs affect tissue blood flow and that blood loss occurs through mechanisms other than blood pressure reduction. The current study, clonidine reduced bleeding through mechanisms other than hypotension. Clonidine was shown to reduce bleeding during middle ear surgery under isoflurane anesthesia in 2 studies. It also reduced the doses of isoflurane and fentanyl in both studies (13–16). In our study, clonidine significantly reduced the dose of remifentanil needed to maintain the same levels of hypotension as the control group. This anesthetic and analgesic-sparing effect is a hallmark of clonidine, as shown in nearly all studies of the effects of clonidine in anesthesia with various agents and for various types of surgery.

The exact mechanism by which controlled hypotension decreases blood loss is still unclear. Some authors have hypothesized that hypotensive anesthesia gives rise to an ischemic wound, which then causes less blood loss. But few studies have attempted to measure blood flow through scientific measures, such as flowmetry (2). Lee *et al.* measured blood flow in the paraspinal muscles during spine surgery with 2 hypotensive drugs, reaching a similar degree of hypotension. They found widely differing values for local blood flow (25), although blood loss did not differ. This result indicates that the effect on local blood flow is not the only factor that is involved. The effect on blood flow in the epidural venous plexus (5) and blood pressure alone (26) have also been hypothesized by studies to influence blood loss. In the context of spinal fusions, some groups report that because bleeding is linked primarily to bone decortication and is, therefore, essentially venous, blood loss will not be influenced by a decrease in arterial pressure (27). As discussed, our study shows that clonidine reduces blood loss; thus, perhaps tissue blood flow occurs through mechanisms other than reductions in blood pressure. Clonidine is an alpha-2 adrenoceptor agonist that effects sedation and antinociception by stimulating central alpha-2 adrenoceptors at different sites in the central nervous system. Stimulation of medullary alpha-2 adrenoceptors decreases sympathetic tone and increases vagal activity, which blunts the hemodynamic responses to stressful stimuli. In addition, stimulation of presynaptic alpha-2 adrenoceptors decreases the release of norepinephrine at peripheral sympathetic nerve endings, which decreases sympathetic tone (28). These mechanisms may be responsible for its hypotensive effects, but it has also been shown to potentiate postjunctional alpha-1 adrenoceptor-mediated vasoconstriction (29–31). The exact mechanism of potentiation of vasoconstriction by clonidine remains unclear. Although Tanaka and Nishikawa attribute this vasoconstrictive action of clonidine to postjunctional alpha-1 adrenoceptor agonism (29), Talke *et al.* suggest that clonidine acts on the alpha-2b subtype of alpha-2 adrenoreceptors in peripheral vascular smooth muscle to cause vasoconstriction (32).

Factors other than blood pressure, postulated to affect intra-operative blood loss include intra-abdominal pressure (related to prone positioning), the number of spinal segments being operated on, body weight, the pathological entity of the disease necessitating surgery (spine surgery due to tumoral lesions is associated with more bleeding), and surgeon’s experience (32). In our study, all patients were operated due to traumatic fractures of the spine on 3 to 4 spinal segment levels and by the same surgical team. There was no significant difference in weight between the two groups, and all patients were positioned in the same way and by the same team. Thus, the effects of the above mentioned factors have been negated.

Our study shows that clonidine, as oral premedication at a dose of 3 μg/kg, is effective in reducing intraoperative blood loss in posterior spinal fusion. It is probably effective in more complicated spine surgeries, such as scoliosis surgery. Also, its effect in reducing blood loss appears to be in part independent of its hypotensive effects. Thus, it is possible that it has the same effect at higher blood pressure, which can obviate the need for hypotensive anesthesia.
